# *Panax notoginseng* Saponins Attenuate Phenotype Switching of Vascular Smooth Muscle Cells Induced by Notch3 Silencing

**DOI:** 10.1155/2015/162145

**Published:** 2015-10-11

**Authors:** Nan Liu, Dazhi Shan, Ying Li, Hui Chen, Yonghong Gao, Yonghua Huang

**Affiliations:** ^1^Department of Neurology, Military General Hospital of Beijing PLA, Beijing 100700, China; ^2^191 Clinical Department, 303 Hospital of People's Liberation Army, Guigang, Guangxi Zhuang Autonomous Region 537105, China; ^3^Key Laboratory of Chinese Internal Medicine of Ministry of Education and Beijing Dongzhimen Hospital, Beijing University of Chinese Medicine, Beijing 100700, China

## Abstract

*Panax notoginseng* saponins (PNS) could maintain vascular smooth muscle cells (VSMCs) in stable phenotypes so as to keep blood vessel elasticity as well as prevent failing in endovascular treatment with stent. Downregulation of Notch3 expression in VSMCs could influence the phenotype of VSMCs under pathologic status. However, whether PNS is able to attenuate the Notch3 silencing induced phenotype switching of VSMCs remains poorly understood. Primary human VSMCs were transfected with a plasmid containing a small interfering RNA (siRNA) against Notch3 and then exposed to different doses of PNS. The control groups included cells not receiving any treatment and cells transfected with a control siRNA. Phenotypic switching was evaluated by observing cell morphology with confocal microscopy, as well as examining *α*-SM-actin, SM22*α*, and OPN using Western blot. Downregulated Notch3 with a siRNA induced apparent phenotype switching, as reflected by morphologic changes, decreased expression of *α*-SM-actin and SM22*α* and increased expression of OPN. These changes were inhibited by PNS in a dose-dependent manner. The phenotype switching of VSMCs induced by Notch3 knockdown could be inhibited by PNS in a dose-dependent manner. Our study provided new evidence for searching effective drug for amending stability of atherosclerotic disease.

## 1. Introduction 

With ageing population worldwide, atherosclerotic disease is responsible for nearly 50% of all deaths [1]. Atherosclerosis is a condition in which plaques build up inside the large and medium-sized arteries. Plaques may partially or totally block the blood flow through the artery system. Some of the diseases may develop as a result of atherosclerosis including stroke, coronary heart disease, and carotid artery disease. Medium-sized arteries may contain up to 40 layers of smooth muscle cells in the media, so they are also called muscular arteries. Phenotypic switching of the vascular smooth muscle cells (VSMCs) is a central pathologic feature in atherosclerosis lesion development, progression, and end-stage disease consequences such as plaque rupture with possible myocardial infarction or stroke. Plaque stability is highly dependent on the VSMCs phenotype, which may either undergo apoptosis or activate the production of matrix metalloproteinases or inflammatory mediators that in turn trigger plaque rupture and thrombosis [2].

Recently, saponins from* Panax notoginseng* (PNS) have been widely used in the treatment of atherosclerotic diseases like stroke and coronary heart disease in China. PNS, the root of* Panax notoginseng*, is a highly valued and important traditional Chinese medicine, which is mainly made up of ginsenoside Rg1, ginsenoside Rb1, notoginsenoside R1, and so forth [3], belonging to the Araliaceae family. Numerous studies have reported that PNS had the therapeutical effect in cardiovascular diseases because PNS could relax blood vessel [4], decrease platelet aggregation [5], and inhibit the inflammatory response in atherosclerotic lesion [6].

Similar to our study, many experiments had proven that PNS may have effect on abnormal proliferation VSMCs to prevent atherosclerosis and restenosis. Some research showed that PNS downregulated the expression of proliferating cell nuclear antigen (PCNA), fibronectin (FN), and matrix metalloproteinase-9 (MMP-9) to inhibit the VSMCs proliferation [7, 8]. Other studies have been focusing on the mechanism of PNS therapeutical effect in the view of signal transduction pathway. For example, Yuan et al. [9] reported that PNS inhibits atherogenesis by suppressing FAK phosphorylation, integrins expression, and NF-*κ*B translocation. Xu et al. [10] also found that PNS inhibits VSMCs proliferation and induces VSMCs apoptosis through upregulating p53, Bax, and caspase-3 expressions and downregulating Bcl-2 expression.

However, few studies described the effects of PNS on the morphology of VSMCs and proteins alteration in pathologic state.

Notch3 is predominantly expressed in adult VSMCs. Notch3 gene mutations cause the cerebral autosomal dominant arteriopathy with subcortical infarcts and leukoencephalopathy (CADASIL), an inherited early stroke syndrome, which results in dementia because of systemic vascular degeneration. This suggests that Notch3 plays a critical role in maintaining the phenotypic stability of vascular smooth muscle cells (VSMCs) [11].

Notch3 mutant VSMCs had marked alterations in shape and size [12]. Mutant VSMCs are thinner and often have thin elongated cytoplasmic processes, as well as phenotype change from “contractile” to “synthetic.” This leads to obvious proliferation of VSMCs and increased secretion of extracellular matrix [13].

Contractile VSMCs are typically characterized by high expression of the genes for contractile elements, including *α*-smooth muscle actin (*α*-SM-actin), smooth muscle 22*α* (SM22*α*), SM myosin heavy chain (MHC), and smoothelin [14, 15], whereas synthetic VSMCs have high expression of osteopontin (OPN), a kind of extracellular matrix proteins secreted by synthetic VSMCs. Therefore, OPN is hardly expressed in contractile VSMCs [16]. Besides, there are some other markers expressed on synthetic VSMCs including epiregulin, tropoelastin, and thrombospondin.

Although these markers are specifically expressed in the fully contractile VSMCs, most of them may be expressed at least transiently in other cells during the tissue repair, or disease stage. Therefore, evidence of expression of a single contractile VSMCs marker gene alone is not sufficient for VSMCs identification and assessment. Thus, the rigorous identification of contractile VSMCs requires examination of multiple marker genes [17]. In order to identify VSMCs phenotype accurately, our study detected the expression of the *α*-SM-actin and SM22*α* markers of contractile VSMCs, as well as the expression of the OPN related to synthetic VSMCs.

In current study, we induced phenotype switching in VSMCs using a siRNA against Notch3 in primary culture of VSMCs. Effects of PNS on phenotype switching were examined by observing cell morphology and the expression of *α*-SM-actin, SM22*α*, and OPN.

## 2. Materials and Methods

### 2.1. Cells and Cell Culture

VSMCs were obtained from human aortic arteries from donors who died of traffic accidents, within 24 h of death, to establish primary culture in DMEM (Invitrogen, Grand Island, NY, USA) containing FBS (Invitrogen) in 5% CO_2_ at 37°C. The acquisition of the samples was approved by the Ethical Board of the General Hospital of Beijing PLA, Beijing, China. All experiments were performed on cells at least 3 times.

### 2.2. Reagents and Antibodies

PureLink DNAse set and PureLink RNA mini kit were purchased from Invitrogen (Grand Island, NY, USA). High-Capacity cDNA Reverse Transcription kit and Power SYBR green Mastermix were purchased from Applied Biosystems (Foster City, CA, USA). Polyvinylidene difluoride (PVDF) membranes were purchased from Bio-Rad (Berkeley, CA, USA). Amersham ECL Plus Western Blotting Detection reagents were purchased from GE Healthcare (Rockford, IL, USA). Xuesaitong injection (containing 400 mg PNS per ampule) was obtained from the Kunming Pharmaceutical Group Co., Ltd. (Yun Nan province, China). Rabbit anti-Notch3, mouse anti-SM22*α*, and rabbit anti-OPN were purchased from Abcam (Cambridge, MA, USA). TRITR-phalloidin, FITC-phalloidin, and mouse anti-*α*-SM-actin were purchased from Sigma-Aldrich (St. Louis, MO, USA). Mouse anti-*β*-actin and mouse anti-*β*-tubulin were purchased from Sigma (St. Louis, MO, USA).

### 2.3. RNA Silencing

To generate adenovirus transfection particles, a set of 4pAd/CMV/V5-DEST vectors (Invitrogen) encoding siRNA targeting the Notch3 gene (Gen Bank accession number NM_000435.2, mRNA, 8089 bp) and a nontarget siRNA control vector (TRC1 library, Sigma-Aldrich, St. Louis, MO, USA) were used to cotransfect HEK293A cells (Invitrogen) using pDONR221 (Invitrogen). VSMCs were seeded onto 6-cm plates (6 × 10^5^ cells per plate) 24 h before transfection. Viral transfection was carried out using a medium containing adenoviruses particles. The culture medium was replaced 24 h later. The transfected VSMCs were selected 48 h after transfection. Stable cell lines created with two vectors (referred to as pAD-EGFP-Notch3-1 and pAD-EGFP-Notch3-3, resp.) showed significant reduction of Notch3 protein expression. Subsequent experiments were performed using stable cell lines generated with the pAD-EGFP-Notch3-1 construct. Nontransfected VSMCs and control siRNA VSMCs had similar growth curves. The details were described in our earlier study and have proven that Notch3 gene decreased significantly [18].

### 2.4. Intervention

Primary culture of VSMCs was transfected with pAD-EGFP-Notch3-1 and treated with PNS (800, 400, or 200 mg/L) or vehicle control. Vehicle control cells, which were transfected with empty plasmid, exhibited no detectable fluorescence. VSMCs transfected with pAD-EGFP-Notch3-1 exhibited a transfection efficiency of more than 50% at 48 h after transfection, called control siRNA. These results had been proven in our earlier study [18]. The control conditions also include blank control and cells treated with the control siRNA only.

### 2.5. Cell Morphology and Immunocytochemistry

VSMCs were fixed with paraformaldehyde for analysis of cell morphology. Fixed cells were stained by FITC or TRITC labeled phalloidin to show actin. Cell images were analyzed and captured with a FluoView 1000 laser scanning confocal microscope (Olympus, Tokyo, Japan).

### 2.6. Western Blot Analysis

Samples of equal amounts of protein were separated by gel electrophoresis, transferred onto polyvinylidene difluoride (PVDF) membranes, and incubated with a primary antibody against one of the following: *α*-smooth muscle actin (*α*-SM-actin), smooth muscle 22*α* (SM22*α*), and osteopontin (OPN), followed by treatment with a secondary antibody. Amersham ECL Plus Western Blotting Detection reagents were used for chemiluminescence. Band intensity was analyzed using the ImageJ analysis software (NIMH, Bethesda, MD, USA) and normalized to *β*-actin and *β*-tubulin as internal control. Protein concentration was determined using a BCA assay (Pierce, Rockford, IL, USA).

### 2.7. Statistics

All experiments were repeated for a minimum of three times. Data were analyzed by one-way analysis of variance (ANOVA) followed by post hoc Dunnett's *t*-test for multiple comparisons. *P* values of <0.05 were considered statistically significant.

## 3. Results

### 3.1. Morphology of VSMCs

**Figure 1:**
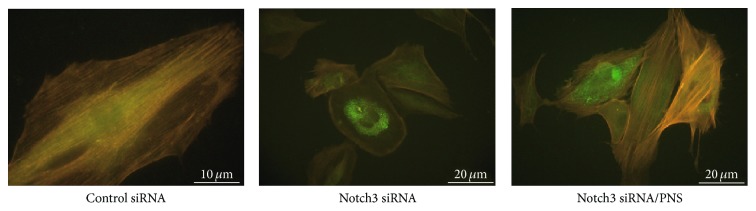
TRITC labeled phalloidin staining showing actin cytoskeleton of VSMCs. Under the excitation wavelength of 488 nm, the cytoskeletons of control and Notch3 siRNA VSMCs were reddish-yellow and the GFP dots were green in the cytoplasm. Notch3 siRNA VSMCs are more rounded and irregular and lack actin cytoskeleton. Upon treatment with PNS, VSMCs became regular.

Morphological characteristics of all VSMCs were tested. Compared to blank control, control siRNA did not affect the morphology or the cytoskeleton pattern. Actin-phalloidin staining revealed normal actin filament system and cell shape (Figure 1). Notch3 siRNA VSMCs with actin-phalloidin staining revealed abnormal nuclear configuration, a disorganized actin filament system, and polygonal cell shape. In addition to decreased cell size, some filaments of F-actin became shrunk and lost (Figure 1). PNS significantly decreased the number of polymorphous cells and increased intercellular gaps and cell size. These findings suggested that the PNS could maintain stable VSMCs phenotypes.

### 3.2. PNS Increased the Expression of *α*-SM-Actin and SM22*α* and Decreased the Expression of OPN in Notch3 siRNA VSMCs

**Figure 2:**
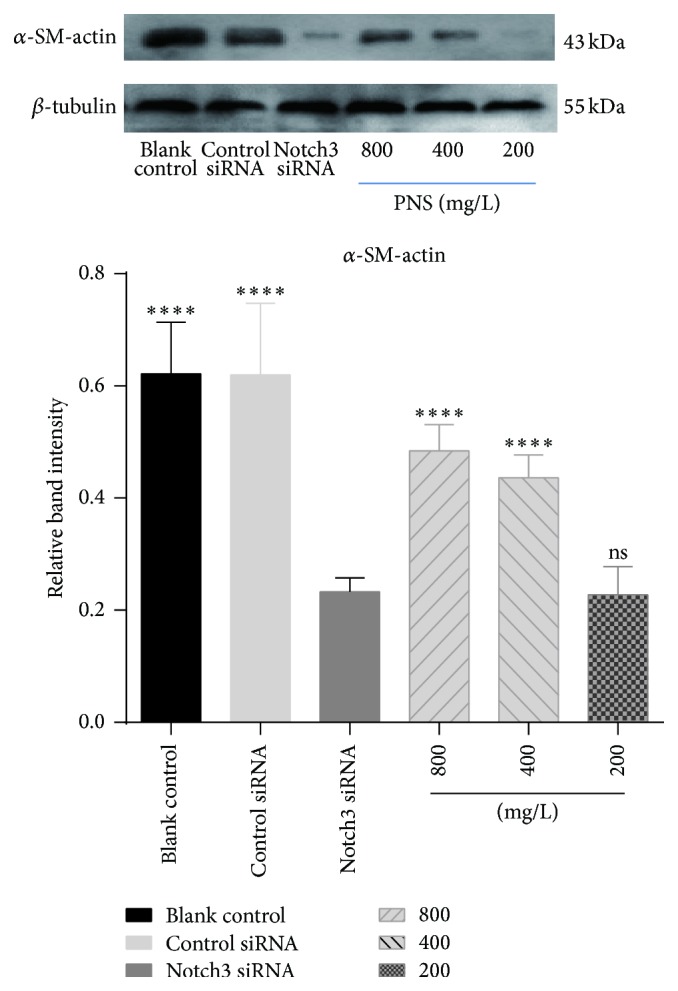
Effects of PNS on *α*-SM-actin in VSMCs transfected with a Notch3 siRNA. *α*-SM-actin was examined with Western blot. The data represent mean ± S.D. ^*∗∗∗∗*^ *P* < 0.0001 versus Notch3 siRNA VSMCs.

**Figure 3:**
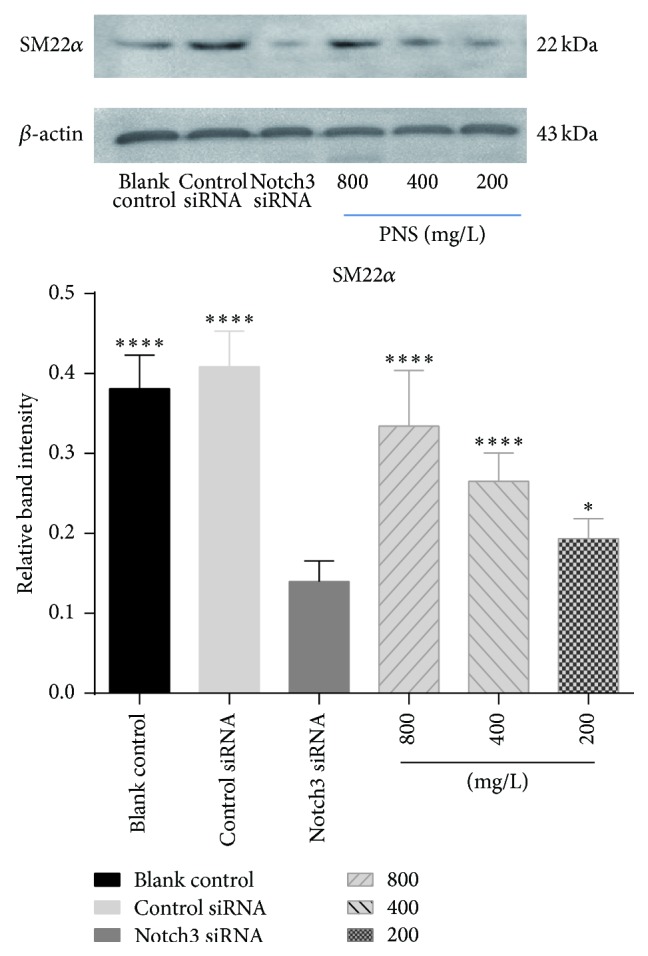
Effects of PNS on SM22*α* in VSMCs transfected with a Notch3 siRNA. SM22*α* was examined by Western blot. The data represent mean ± S.D. ^*∗∗∗∗*^ *P* < 0.0001 or ^*∗*^ *P* < 0.05 versus Notch3 siRNA VSMCs.

The relative expression levels of *α*-SM-actin and SM22*α* protein in blank control group were 0.622 ± 0.088 and 0.381 ± 0.040, 0.620 ± 0.123 and 0.409 ± 0.043 in cells exposed to the control siRNA, and 0.233 ± 0.023 and 0.140 ± 0.025 in cells treated with Notch3 siRNA, respectively. PNA treatment of the cells transfected with Notch3 siRNA at 800, 400, and 200 mg/L increased relative expression levels of *α*-SM-actin and SM22*α* expression to 0.485 ± 0.044, 0.436 ± 0.040, 0.228 ± 0.048 and 0.335 ± 0.067, 0.265 ± 0.0341, 0.194 ± 0.024, respectively. The relative expression levels of *α*-SM-actin and SM22*α* protein of VSMCs were positively correlated with PNS concentration (Figures 2 and 3). In comparison to cells transfected with Notch3 siRNA, PNS at 400 and 800 mg/L, but not 200 mg/L, significantly upregulated protein expressions of *α*-SM-actin (*P* < 0.0001). Similar to *α*-SM-actin, PNS significantly upregulated protein expressions of SM22*α* in a dose-dependent manner (*P* < 0.05 or 0.01). These results suggested that PNS could maintain *α*-SM-actin and SM22*α* protein level and stabilize the VSMCs phenotype at high concentration.

OPN protein was not detectable in blank control group or transfected with the control siRNA group. Notch3 siRNA increased the OPN protein to 0.735 ± 0.107. PNS treatment decreased the OPN protein to 0.379 ± 0.069, 0.486 ± 0.048, and 0.691 ± 0.070 at 800, 400, and 200 g/mL. In comparison with cells transfected with Notch3 siRNA, PNS at 400 and 800 mg/L, but not 200 mg/L, significantly downregulated protein expressions of OPN (*P* < 0.0001).

**Figure 4:**
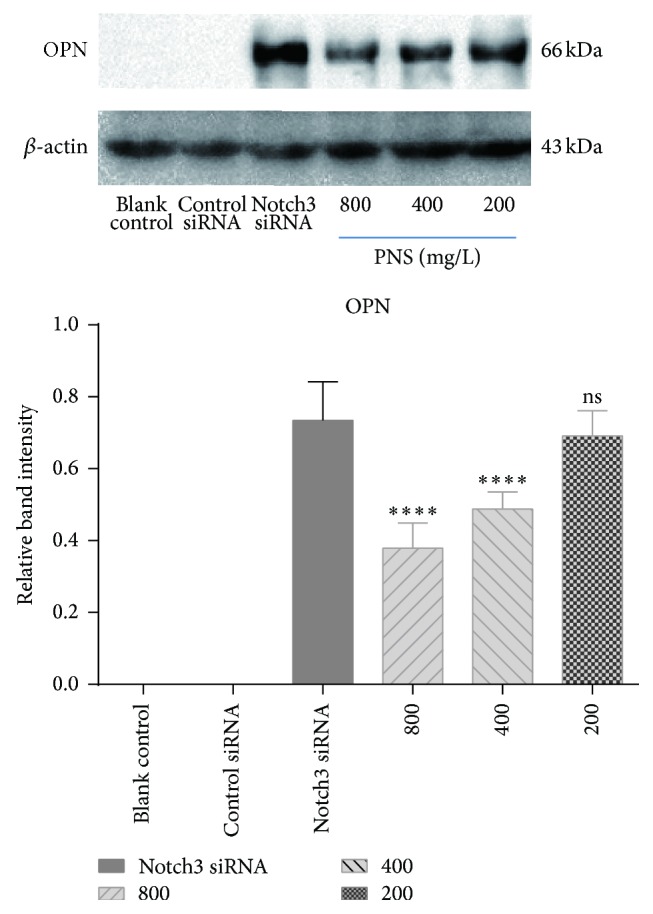
Effects of PNS on OPN in VSMCs transfected with a Notch3 siRNA. OPN was examined with Western blot. The data represent mean ± S.D. ^*∗∗∗∗*^ *P* < 0.0001 versus Notch3 siRNA VSMCs.

PNS may inhibit OPN overexpression and stabilize the VSMCs phenotype too (Figure 4).

## 4. Discussion

Control of VSMCs phenotype is essential in the development and maintenance of a healthy vasculature. The contractile phenotype can be altered by phenotypic modulation leading to a high rate of proliferation and migration and extracellular matrix (ECM) accumulation while markers of VSMC contractility are downregulated. Notch3 protein, a large single pass transmembrane receptor, is exclusively expressed almost in VSMCs and pericytes, the specialized VSMCs, but is not detectable in endothelial cell which is important for survival of VSMCs and plays a critical role of Notch3 for VSMCs in blood vessel integrity and blood-brain barrier function in the mammalian vasculature [2]. Notch3 is the first cell-autonomous regulator of arterial differentiation and maturation of VSMCs. In vivo studies have shown that Notch3−/− vessels lost their arterial phenotype, from contractile to synthetic, indicating that Notch3 is crucial for the phenotypic integrity of VSMCs [19]. There is growing evidence for a pivotal role of VSMC plasticity and phenotypic switching in vascular diseases, such as atherosclerosis, vein graft stenosis, and restenosis following angioplasty and stenting [20]. In our study, using the RNA interference approach, we studied the role of Notch3 in the phenotype switch of VSMCs. In order to tackle this problem, we do some work from two aspects: morphological pattern observed by laser scanning confocal microscope and the marker proteins of VSMCs detecting by Western Blot.

In physiological state, VSMCs are large and elongated and have abundant cytoskeleton and prominent nucleus. In culture, these cells orient themselves into parallel bundles and become spindle-shaped. Notch3 siRNA VSMCs, lacking actin cytoskeleton, change their shape from elongated to more rounded and irregular [21]. These cells have more pseudopods. This is identical to our observation (Figure 1). However, the morphology of Notch3 siRNA VSMCs with PNS was better than control cells. Their shapes were regular without much pseudopods. Then we further investigated the cytoskeleton and ECM related to VSMCs phenotypic switching. *α*-SM-actin and SM22*α* are considered VSMC-specific contractile proteins as well as important cytoskeletal proteins. OPN is one of the synthetic VSMCs markers and a multifunctional protein in ECM. It is hardly expressed by contractile VSMCs. VSMCs in the blank control treated with the control siRNA only maintain the contractile phenotype and hardly expressed OPN. This is consistent with our result in Western blot (Figure 4).

VSMCs phenotype switching from contractile to synthetic is a key step in which VSMCs revert to an immature, proliferative phenotype, leading to pathological luminal narrowing. This process involves a reduction in the expression of the VSMCs contractile proteins such as *α*-SM-actin and SM22*α* that are the hallmarks of quiescent, mature VSMCs [22]. Meanwhile, the expression level of OPN increases gradually. It has been reported that secretory calcitonin gene-related peptide (CGRP) and CGRP receptor modified mesenchymal stem cells on proliferation and phenotypic transformation of VSMCs. They found that the expression of contractile phenotype protein *α*-SM-actin declined while intermediate phenotype OPN increased significantly [23]. However, only two markers proteins seemed to be insufficient to support phenotypic changes. Another study showed that *α*-SM-actin and calponin protein levels significantly decreased by OPN overexpression. Downregulation of *α*-SM-actin and calponin was also observed on extracellular treatment of mouse VSMCs with recombinant OPN [24]. It is worth noting that our study found that there is a negative relation between *α*-SM-actin, SM22*α*, and OPN, which has never been reported before. And we observed that PNS could increase the expression of contractile markers *α*-SM-actin and SM22*α* and decrease the expression of synthetic marker OPN in a dose-dependent manner. PNS at concentration of 200 mg/L only increased the expression of SM22*α* in control cells and had little influence on the expression of *α*-SM-actin and OPN. This suggested PNS at high concentration of 400 and 800 mg/L had a better protective effect than that at low concentration of 200 mg/L.

## 5. Conclusions 

Notch3 plays an important role in the phenotype switch of VSMCs and may represent a target for treatment of atherosclerotic diseases. We showed improved morphology by PNS in VSMCs treated with a Notch3 siRNA: the polymorphous cells became regular with PNS treatment. Notch3 siRNA transfection decreased *α*-SM-actin and SM22*α* and increased OPN. PNS attenuated these changes, particularly at high concentration range. These results suggested that PNS could maintain VSMCs contractile phenotype and encourage exploring of the therapeutic potentials of PNS in vascular diseases.

A few limitations of this study need to be mentioned. First, the migration ability of VSMCs was only assessed morphologically. Secondly, this study focused on the phenotype switch of VSMCs by detecting marker proteins. Future studies are needed to address signal pathways through that phenotype switch of VSMCs.
